# The role and mechanism of macrophage autophagy in the experimental model of chronic obstructive pulmonary disease

**DOI:** 10.18332/tid/186403

**Published:** 2024-04-23

**Authors:** Li Zhang, Tian Cheng, CaiHong Liu, ShengYang He, JunJuan Lu

**Affiliations:** 1 Department of Respiratory and Critical Care Medicine, The Third XiangYa Hospital of Central South University, Changsha, China; 2 Department of Respiratory and Critical Care Medicine, The Second XiangYa Hospital of Central South University, Ghangsha, China

**Keywords:** chronic obstructive pulmonary disease, macrophage autophagy, ATG5^myeΔ^, cigarette smoke extract

## Abstract

**INTRODUCTION:**

Macrophages play an important role in chronic obstructive pulmonary disease (COPD). Cigarette smoke (CS) impairs autophagy in alveolar macrophages from COPD patients, and autophagic impairment leads to reduced clearance of protein aggregates, dysfunctional mitochondria, and defective bacterial delivery to lysosomes. However, the exact function of lung macrophage autophagy in the pathogenesis of CS-induced COPD remains largely unknown.

**METHODS:**

Western blot detected the expression of autophagy-related proteins induced by CSE. The model of COPD mice was established by CS exposure combined with CSE intraperitoneal injection. Double immunofluorescence was used to measure the CD206+LC3B+ cells. The morphological changes and effects on lung function were observed. Masson staining detected the changes in collagen fibers in lung tissue. The expression levels of E-cadherinb and N-cadherinb were detected by immunohistochemistry. Western blot detected the expression of ATP6V1E1 in lung tissue.

**RESULTS:**

At 24 hours of exposure to CSE, the expression levels of LC3B (microtubule-associated protein 1A/1B-light chain 3B) and P62 (nucleoporin 62) were highest at 1% CSE and AGT5 (nucleoporin 62) at 2.5% CSE; at 48 hours, the expression levels of LC3B, P62 and AGT5 were highest at 2.5% CSE, and as the intervention time increased.CD206+LC3B+ cells were significantly higher in the COPD group. Enhanced macrophage autophagy may promote emphysema formation and aggravate lung function damage. The expression of E-cadherinb in lung tissue of the COPD group was decreased, and N-cadherinb expression was increased; the expression of E-cadherinb was increased, and N-cadherinb expression was decreased in ATG5^myeΔ^ COPD mice. The expression of ATP6V1E1 in the lung tissue was increased in the COPD group; ATP6V1E1 expression was decreased in the lung tissues of ATG5^myeΔ^ COPD mice.

**CONCLUSIONS:**

CSE enhanced macrophage autophagy, leads to increased lung function impairment and collagenous fiber in lung tissue, as well as promotes epithelial-mesenchymal transition, and eventually leads to small airway remodeling, which may be achieved through the ATG5/ATP6V1E1 pathway.

## INTRODUCTION

Over 100 million people are living with chronic obstructive pulmonary disease (COPD), which is the third leading cause of death in the world^1,2^. In this disease, pulmonary macrophages, neutrophils, and lymphocytes are prevalent in the airways, lung parenchyma, and pulmonary vasculature, causing persistent inflammation and lung destruction^3^. It has been shown that lung macrophages play a key role in clearing inflammatory mediators, maintaining lung homeostasis, and influencing COPD pathogenesis^4,5^. It has been found that COPD is associated with an elevated number of lung macrophages and impaired function of these macrophages, which may contribute to disease pathogenesis. The smoking habit is a major contributing factor to chronic obstructive pulmonary disease, leading to chronic inflammation of the airways, mucus hypersecretion, tissue destruction, and remodeling of the small airways^6,7^. However, the cellular and molecular mechanisms mediating the pathogenesis of cigarette smoke (CS)-induced COPD remain largely unknown. Autophagy is responsible for the turnover of organelles and long-lived proteins in a dynamic process that occurs within cells every few seconds. It plays a crucial role in maintaining intracellular homeostasis and adapting to adverse circumstances^8^. However, autophagy can also cause cellular damage and accelerate disease progression^9-11^. LC3B-/- (microtubule-associated protein 1A/1B-light chain 3) mice seem immune to many pathological features associated with smoking, whereas COPD lung tissue contains increased autophagosome numbers and LC3 expression^12-14^. Similar results were observed in bronchial epithelial cells when cigarette smoke extract (CSE) enhanced the expression of LC3B-II and induced autophagy, while knockdown of autophagy proteins LC3B and Beclin-1 protected against CSE-induced apoptosis^15^. CS’s impairing autophagy impairs alveolar macrophages from COPD patients, and, as a result, protein aggregates, dysfunctional mitochondria, and defective bacterial delivery to lysosomes are less likely to be cleared^16^. However, the exact function of lung macrophage autophagy in the pathogenesis of CS-induced COPD remains largely unknown. The present study investigates the effect of CSE on macrophage autophagy and also the possible role and mechanisms of macrophage autophagy in COPD.

## METHODS

### Animals, preparation of CSE and animal model

ATG5^myeΔ^ mice were constructed concerning the previously described method^17^, and C57BL/6 and ATG5^myeΔ^ C57BL/6 mice were randomly divided into control and COPD groups, respectively, all of which were purchased from GemPhramatech. The preparation of CSE was performed according to the previously described procedure^18^. The COPD mouse model was established by CS exposure combined with intraperitoneal injection of CSE. The duration of modeling was 28 days. Among them, on days 1, 12, and 23, the normal control and ATG5^myeΔ^ groups were injected intraperitoneally with PBS at 0.3 mL PBS per 20 g of body weight; the wild COPD and ATG5^myeΔ^ COPD groups were injected intraperitoneally with CSE at 0.3 mL CSE per 20 g of body weight; the modeling cassettes were prepared as described previously^19^. Five cigarettes were lit simultaneously for 15 minutes, followed by opening the box and allowing the animals to rest for five minutes, followed by a repeat of the smoking, with exposure twice a day. On day 29, the lung function of the mice was measured, and lung tissue was collected. The study was approved by the CSU Institutional Review Board and followed the guidelines for animal and human studies^20^.

### Cell culture

Cell culture and production of the RAW264.7 cell line was purchased from the American Type Culture Collection and cultured using DMEM (Life Technologies, Carlsbad, CA, USA) containing 10% (v/v) heat-inactivated fetal bovine serum.

### Pulmonary function measurement

Pulmonary function was measured using a small animal spirometer (PLY3211 system; Buxco Electronics, Sharon, CT, USA), simply by weighing each mouse, followed by anesthesia with 1% sodium pentobarbital (0.2 mL/20 g BW) for intraperitoneal anesthesia, followed by tracheotomy, tracheal intubation, and intubation connected to a computer-controlled small animal spirometer. Airway resistance (Raw), pulmonary kinetic compliance (Cdyn), peak expiratory flow (PEF), and inspiratory time/expiratory time (Ti/Te) were measured.

### Histomorphology of lung tissue

The lung was lavaged with 4% paraformaldehyde and then fixed overnight in 4% paraformaldehyde. Lung tissue was embedded in paraffin (Sigma, MO, USA), cut into 4 μm sections, and stained with hematoxylin and eosin (H&E) and Masson’s trichrome staining solution (Sigma). Finally, each section was observed with a light microscope (BX41, Olympus, Japan), and the Masson-stained collagen volume fraction (CVF) was measured using Image J.

### Immunohistochemistry (IHC)

Dewaxed and descaled sections were incubated with 1% H2O2 for 30 minutes at room temperature to eliminate endogenous peroxidase activity, then anti-N-cadherin and anti-E-cadherin antibodies (PTG, California, USA) were added and incubated overnight at 4°C. On day two, the sections were thoroughly washed and incubated with secondary antibodies for 1 hour at room temperature. The secondary antibody needs to be coupled with the appropriate horseradish peroxidase. Unreacted secondary antibodies were removed, and sections incubated with 3,39-diamino biphenyl-4HCl (DAB; Sigma, MO, USA)-H2O2 solution, observed for immunolabelling and then sections re-stained with H&E using fixed glass covers. The presence of N-cadherin and E-cadherin can be indicated by varying degrees of tan or brown granules or flaky deposits. Four to five positive N-cadherin and E-cadherin images were randomly selected, and the integrated optical value (IOD) was calculated. High magnification of each section was performed using Image-ProPlus version 6.0 (Media Cybernetics, Inc., Rockville, MD, USA) software to visualize high-resolution color pathology images and analyze the results.

### Double immunofluorescence

Lung sections were washed with phosphate-buffered saline containing 0.3% TritonX-100 (PBST) and then incubated in 5% horse serum for 30 minutes to block the nonspecific binding of the antibodies. Next, sections were incubated with two different primary antibodies (Table 1) overnight at 4°C. After incubation, the primary antibodies were washed away with PBST, and sections were incubated with the corresponding fluorescent secondary antibodies (Table 2) for 2 h at room temperature. Tissue sections were stained with 4-diamino-6-diamino-2-phenylenediamine hydrochloride (DAPI) for 10 min and then washed with PBST. Tissue sections were observed using a laser confocal microscope (Leica, Germany), and the stained images were processed by the corresponding software.

**Table 1:** Double immunofluorescence primary and secondary antibody dilution ratio

*Reagents*	*Manufacturers*	*Dilution ratios*
(primary antibodies) CD206+LC3	Proteintech Group	1:800+1:100
(secondary antibody) CY3 goat anti-mouse	Servicebio	1:300
(secondary antibody) 488 Goats against rabbits	Servicebio	1:400

### Western blot

Lung tissues and cells were homogenized manually in a glass homogenizer and lysed on ice in RIPA lysis solution for 30 min. Protein assays were performed using the Bicinchoninic Acid Assay (BCA) protein quantification kit (Wellbio Inc, Changsha, China). Proteins (30–60 μg) were mixed 1:1 with two sodium dodecyl sulfate (SDS) buffer (20% glycerol, 4% SDS, 3.12% dithiothreitol, 0.2% bromophenol blue and 0.1 mol/L Tris-HCl, pH 6.8, all from Sigma) and incubated for 4 min at 100°C. Equal amounts of protein from each sample were incubated with 10% SDS -polyacrylamide gel run at 120 V for 90 min and blotted into polyvinylidene fluoride microporous membranes (Microporous, Billerica, MA, USA). Membranes were incubated with diluted ATP6V1E1 (Abcam, London, UK) overnight at 4°C , then washed three times with triphasic buffered saline with Tween (TBS-T), secondary anti-rat antibodies (1:3000; 1 h) were displayed with horseradish peroxidase couples, and then washed again with TBS-T. Immunoreactive bands were developed using ECL chemiluminescent substrates (Thermo, Waltham, MA, USA).

### Statistical analysis

All data were analyzed using SPSS 22.0 statistical software (IBM Corporation, USA) and described as mean and standard deviation. Statistical differences between groups were tested using the non-parametric Mann-Whitney U test and one-way ANOVA. A p<0.05 was considered a statistically significant difference.

## RESULTS

### Effect of CSE on autophagy in RAW264.7 cells

**Figure 1:**
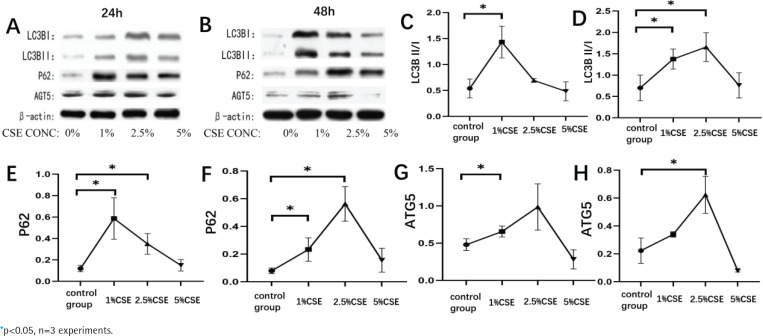
Effect of cigarette smoke extract on RAW264.7 cells autophagy. RAW264.7 cells were treated with different concentrations of cigarette extract for 24 hours and 48 hours, and observed the effects of CSE concentrations and intervention time on the expression of autophagy related proteins (LC3B, P62, ATG5) in RAW264.7 cells. A, C, E and G: Changes in autophagy-related protein expression in RAW264.7 cells at different concentrations of cigarette smoke extract for 24 hours. B, D, F and H: Changes in autophagy-related protein expression in RAW264.7 cells at different concentrations of cigarette extracts for 48 hours

Cigarette smoke is a major risk factor for the development of COPD. In this study, we investigated the effect of cigarette smoke extract on autophagy in RAW264.7 cells. We found that the expression of autophagy-related proteins LC3B, P62, and AGT5 increased significantly after CSE intervention in RAW264.7 cells compared with normal controls, which was statistically significant (p<0.05, Figures 1A and 1B). The expression levels of LC3B, P62, and AGT5 increased with CSE concentration increased, firstly increasing and then decreasing. At 24 hours of intervention with CSE, the expression levels of LC3B and P62 were highest at 1% CSE and AGT5 at 2.5% CSE (p<0.05, Figures 1C, 1E and 1G); at 48 hours of intervention with CSE, the expression levels of LC3B, P62, and AGT5 were highest at 2.5% CSE, and as the intervention time increased, the AGT5 expression decreased and LC3B and P62 expression were not significantly correlated (p<0.05, Figures 1D, 1F and 1H).

### Changes in the number of M2-type macrophage-associated autophagy-positive cells in COPD

In this study, we detected the changes in the number of M2-type macrophage-associated autophagy-positive cells in lung tissues of mice from control group and COPD model group by double immunofluorescence method, using CD206 to label M2 macrophages, LC3B to label autophagy-associated protein, and found that CD206+LC3B+ cells were significantly higher in the COPD group compared with the control group, which was statistically significant (p<0.05, Supplementary file Figures 2A and 2B), indicating that M2 macrophages are closely related to autophagy.

### Effects of macrophage autophagy on lung morphology and lung function in COPD mice

The pathogenic mechanism of macrophage autophagy in COPD is not fully understood. In this study, the COPD model was constructed by constructing specific macrophage knockout mice while using fumigation combined with intraperitoneal injection of liquid CSE, as shown by HE staining results, compared with the control group, histopathological sections of the lungs of the wild COPD group and ATG5^myeΔ^ COPD group showed broken and fractured alveolar walls, forming larger cavities, showing noticeable emphysematous changes, which is consistent with the lung histopathological changes of COPD disease model, and no significant differences were observed morphologically between the control and ATG5^myeΔ^ groups, and the lung inflammatory cell infiltration was increased in ATG5^myeΔ^ COPD mice compared with the wild-type COPD group mice (Supplementary file Figure 3A). Meanwhile, we measured lung function in the four groups of mice and found that Raw increased (p<0.05, Supplementary file Figure 3B) and Cydn, PEF, and Ti/Te decreased (p<0.05, Supplementary file Figure 3B) in the COPD group compared to the normal control group; no significant differences were observed between the control group and ATG5^myeΔ^ group; compared to the wild-type COPD group mice, Raw in ATG5^myeΔ^ COPD mice decreased (p<0.05, Supplementary file Figure 3B) and Cydn, PEF, and Ti/Te increased (p<0.05, Supplementary file Figure 3B), suggesting that enhanced macrophage autophagy may promote emphysema formation and aggravate lung function damage and that emphysema and lung function could be improved after reducing macrophage autophagy.

### Effect of macrophage autophagy on small airway remodeling in COPD

By Masson staining, we found that collagen fibers were significantly increased in the lung tissue of COPD group mice, and there was a decrease in collagen fibers after specific knockdown of macrophage autophagy-related genes, and collagen fibers were increased in the lung tissue of COPD group mice compared with a normal control group (p<0.05, Supplementary file Figures 4A and 4B); no significant difference was seen between the control group and ATG5^myeΔ^ group; compared with wild-type COPD group, lung tissue collagen fibers were reduced in ATG5^myeΔ^ COPD mice (p<0.05, Supplementary file Figures 4A and 4B), suggesting that lung tissue fibrosis may be improved after reducing macrophage autophagy.

Epithelial-mesenchymal transition is one of the important mechanisms of small air remodeling. In this study, E-cadherin was used as an epithelial marker, and N-cadherin as a mesothelial marker, and the expression of E-cadherin and N-cadherin was measured using immunohistochemistry, and it was found that compared with the normal control group, the expression of E-cadherin in lung tissue of COPD group was decreased (p<0.05, Supplementary file Figures 5A and 5B) and N-cadherin expression was increased (p<0.05, Supplementary file Figures 6A and 6B); no significant difference was observed between the control group and ATG5^myeΔ^ group; compared to the wild-type COPD group, the expression of E-cadherin was increased (p<0.05, Supplementary file Figures 5A and 5B) and N-cadherin expression was decreased in the ATG5^myeΔ^ COPD mice (p<0.05, Supplementary file Figures 6A and 6B); the above suggests that increased macrophage autophagy may lead to epithelial-mesenchymal transition in COPD.

### Effect of macrophage autophagy on ATP6V1E1 expression in COPD lung tissue

We found that the expression of ATP6V1E1 in the lung tissue was increased in the COPD group compared with normal controls by Western blot (p<0.05, Supplementary file Figure 7); no significant difference was seen between the control group and ATG5^myeΔ^ group; compared with the wild-type COPD group, ATP6V1E1 expression was decreased in the lung tissues of ATG5^myeΔ^ COPD mice (p<0.05, Supplementary file Figure 7), suggesting that ATP6V1E1 may be an important factor in the development of COPD due to macrophage autophagy.

## DISCUSSION

Our study shows that cigarette extract promotes macrophage autophagy, which is associated with collagen deposition and epithelial-mesenchymal transformation, thus playing a role in small airway remodeling in COPD, and the ATG5/ATP6V1E1 pathway may be involved. Macrophages play an important role in the pathogenesis of COPD. The number of alveolar macrophages in the airways, lung parenchyma, alveolar lavage fluid, and induced sputum is significantly increased in COPD patients, and the number of macrophages in the lung tissue and alveolar cavity is increased up to 25-fold in patients with emphysema compared with controls who smoke but have normal lung function. The number of macrophages is positively correlated with the severity of COPD^21^. In our previous study^22-24^, we found that the proportion and number of M2-type macrophages were significantly increased in COPD compared with the control group and were associated with lung function impairment and lung parenchymal destruction, but there was no similar study on the interaction between M2-type macrophages and macrophage autophagy in COPD. Autophagy, or the process of self-consumption, is the maintenance of internal environmental homeostasis through the degradation of damaged organelles and proteins by lysosomes. After autophagosome formation, the extension of the autophagosome requires two ubiquitin-like binding systems, namely LC3 and the Atg8 and Atg5-Atg12-Atg16L binding systems^25^. Atg7 and Atg10 bind Atg5 to Atg12, and then the Atg5-Atg12 conjugate non-covalently interacts with Atg16L interacts to form a large multimeric Atg16L complex^26^. At the same time, LC3 is cleaved to LC3-I by Atg4 at its C-terminus, whereby Atg7 and Atg3 splice phosphatidylethanolamine (PE) to residues of LC3-I on the C-terminal glycine to produce LC3-II. LC3-II then attaches to both sides of the phagocytic membrane, removing it from the outer membrane before the autophagosome fuses with the lysosome^27^. This process requires the Atg16L complex to define the site of LC3 lipidation and to act in concert with LC3 lipidation to prolong and close the phagocyte^26^. After autophagosome closure, selective autophagy is performed with the ubiquitin-binding protein SQSTM1/p62 on the surface of the autophagosome as the targeting adapter protein, and the autophagosome matures and is directed towards the lysosome^28^. Thus, during autophagy, LC3B, AGT5, and P62 are involved as major autophagic proteins. Autophagy can play a role in attenuating the inflammatory response by inhibiting the production of complexes that promote the inflammatory response and by removing damaged organelles from the cell^29^. Cigarette smoke activates oxidative stress and chronic inflammatory responses because it contains a large number of chemical complexes and oxidative radicals, and persistent chronic inflammation damages alveolar and even bronchial structures within lung tissue, resulting in irreversible damage to lung function and leading to COPD in which autophagy is involved^30^. Meanwhile, in *in vitro* experiments, we used RAW264.7 macrophages with different concentrations of CSE for 24 and 48 hours. We found that the expression of LC3B, P62, and AGT5 increased with increasing concentrations of CSE, which was statistically significant and showed a first increasing and then decreasing, and when the concentration of CSE was 5%, the expression of autophagy decreased compared to the previous one, and we speculated that, due to the high concentration of cell activity was affected, thus affecting the expression of autophagy. When the intervention was 24 h, the expression levels of LC3B and P62 were highest at 1% CSE, and AGT5 was highest at 2.5% CSE. At 48 h of intervention, the expression levels of LC3B, P62, and AGT5 were highest at 2.5% CSE, and with the extension of the intervention time, the expression of AGT5 decreased, and the expression of LC3B and P62 did not correlate significantly, so CSE intervention could increase macrophage autophagy. However, there is a dynamic process of autophagy, and whether its changes fluctuate with the duration of cigarette smoke stimulation needs to be clarified in future experiments by further establishing mouse models of COPD with different durations of cigarette smoke intervention.

Our previous study showed that the proportion of M2 macrophages is increased in COPD and that M2 macrophages may be closely related to lung function impairment and small airway remodeling^31^. In this study, immunohistochemistry revealed that the number of CD206+ LC3B+ cells was significantly increased in the COPD group compared to the control group, but the exact interaction between M2 macrophages and macrophage autophagy has not been elucidated in other studies, which needs to be further elaborated in future experiments. In the pathogenesis of COPD emphysema, which is thought to be due to smoking-induced epithelial cell death, autophagy is involved in regulating apoptosis^32^, and autophagy also plays a role in controlling cell proliferation. A related experimental study showed that the use of azithromycin led to autophagy in airway smooth muscle cells and also inhibited cell proliferation, which in turn had a reparative effect on airway remodeling, while the use of 3-methyladenine, an inhibitor of autophagy, resulted in a significant improvement in cell proliferation^33^. Meanwhile, studies on extracellular vesicles have found that autophagy is involved in regulating cell differentiation. miR-210 in extracellular vesicles directly targets and inhibits the expression of autophagy gene 7, which regulates the control of autophagy and cell differentiation in fibroblasts, and that the formation of extracellular vesicles in bronchial epithelial cells is reduced, thereby inhibiting the role of extracellular vesicles in autophagy of fibroblasts and their conversion to myofibroblasts, leading to airway remodeling in COPD. The above studies suggest that autophagy is involved in the process of small airway remodeling and plays an important role, but the role of macrophage autophagy in COPD has not been described in other studies. After reducing macrophage autophagy, emphysema and lung function could be improved. Compared with wild-type COPD mice, ATG5^myeΔ^ COPD mice showed a reduction in collagen fibers and a possible improvement in lung fibrosis. Small airway remodeling is one of the important pathophysiological processes leading to the development of COPD, and the epithelial-mesenchymal transition is one of the important mechanisms leading to small airway remodeling in COPD, as confirmed by our previous study^31^. Compared with wild-type COPD mice, the expression of E-cadherin increased, and N-cadherin decreased in lung tissues of ATG5^myeΔ^ COPD mice, and the increased autophagy of macrophages may lead to epithelial-mesenchymal transition in COPD lung tissues. The above suggests that macrophage autophagy plays an important role in small airway remodeling in COPD.

Macrophage autophagy may play an important role in the development of COPD, but the exact mechanism is unknown. ATG5 specifically reduces the acidification of exosome-producing late nuclear endosomes by moving the regulatory component ATP6V1E1 into exosomes and disrupting the acidified v1v0-ATPase^33^, suggesting a link between ATG5 and ATP6V1E1, and the role of ATP6V1E1 in COPD has not been studied. It was found that the autophagy proteins ATG5 and LC3 synergistically regulate MVB (multivesicular bodies) acidification and exosome production by controlling the interaction of ATP6V1E1 with the V1V0-ATPase complex^34^. Furthermore, EV (extracellular vesicle) biogenesis and autophagy are thought to be functionally linked as late nuclear endosomes can fuse with immature autophagosomes^35,36^. Our study found that lung tissue ATP6V1E1 expression was increased in COPD mice compared to normal controls and decreased in ATG5^myeΔ^ COPD mice compared to wild-type COPD mice, and we hypothesize that macrophage autophagy may contribute to COPD development by affecting exosomes through ATG5/ATP6V1E1. This needs to be further investigated and elaborated on in future experiments.

### Limitations

Our study has some limitations. First, the mechanism of ATG5/ATP6V1E1 in COPD was not studied in depth. Second, our study was only a phenomenological study. More in-depth and comprehensive research on the mechanism needs to be carried out in the following studies, for example, the interaction and communication relationship between macrophage autophagy and bronchial epithelial cells and other cells.

## CONCLUSIONS

CSE may lead to enhanced autophagy of macrophages, which may aggravate lung function impairment, an increase of lung tissue collagen fibers, epithelial-mesenchymal transformation, and eventually lead to small airway remodeling, which may be achieved through the ATG5/ATP6V1E1 pathway.

## Supplementary Material



## Data Availability

The data supporting this research are available from the authors on reasonable request.
