# Supplementary material for: The role and mechanism of macrophage autophagy in the experimental model of chronic obstructive pulmonary disease
Source: Tob Induc Dis. 2024 Apr 23;22:10.18332/tid/186403. doi: 10.18332/tid/186403 (PMC11037029; doi:10.18332/tid/186403)
Supplement: Supplementary file 1 [file TID-22-67-s1.pdf]

Supplementary Figures

Figure2:

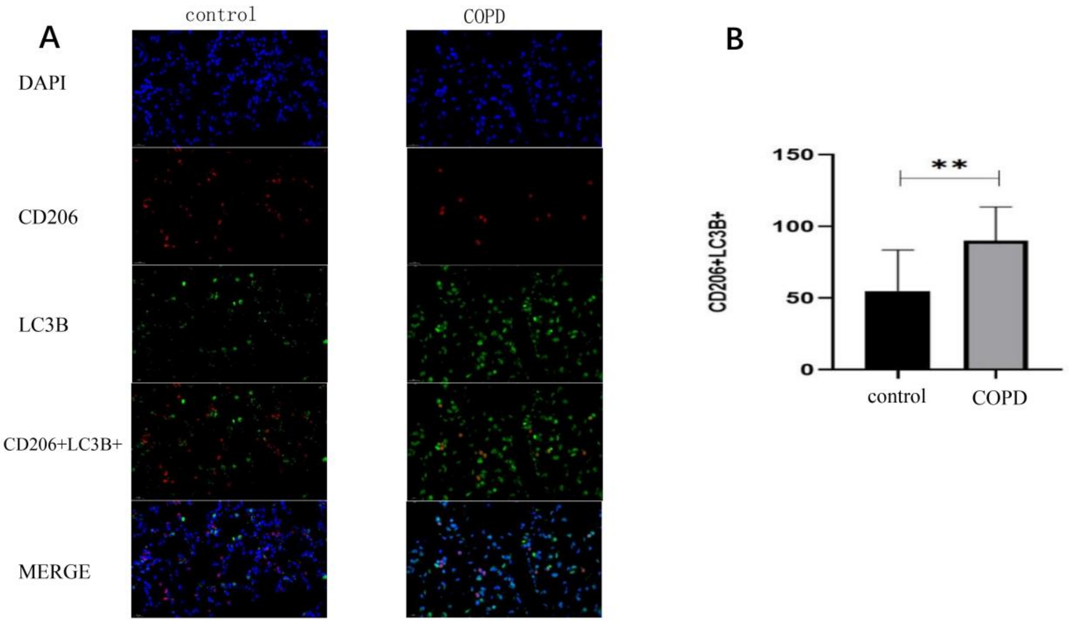

Figure 2: Changes in the number of M2-type macrophage-associated autophagy-positive cells in lung tissues of mice from control group and COPD model group.The number of CD206+LC3B+ double positive cells was determined by dual immunofluorescence method. CD206 was used as the marker of M2 macrophage and LC3B was used as the marker of autophagy. A: Double immunofluorescence staining of labeled lung tissue, using CD206 to label M2 macrophages, LC3B to label autophagy-associated protein (400×magnification). B: Changes in the number of CD206+LC3B+ cells in the control and COPD model groups. n=3 experiments, **P < 0.05.

Figure3:

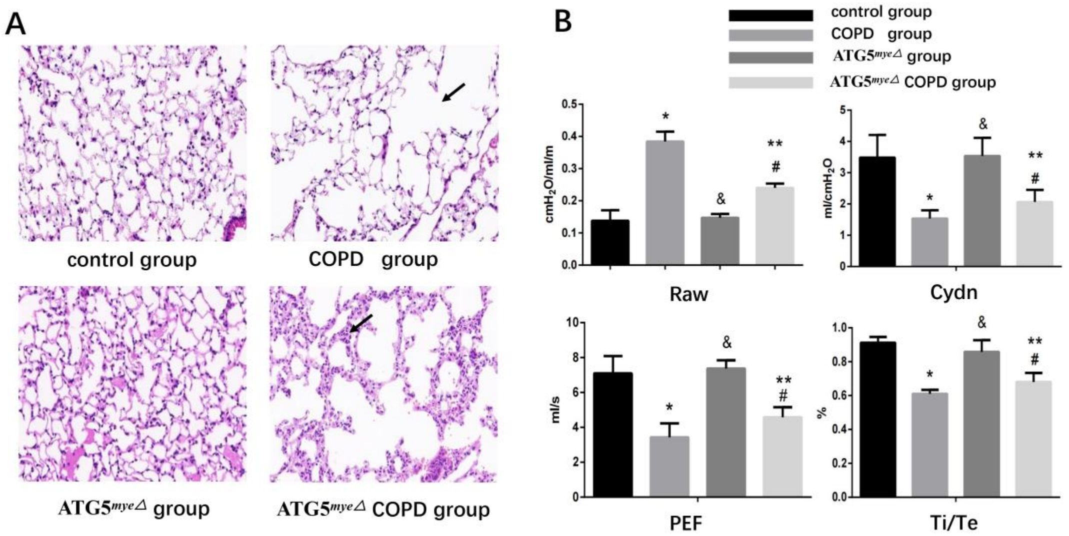

Figure 3: Effects of macrophage autophagy on lung morphology and lung function in COPD mice. Construction of ATG5 $^{mye\triangle}$mice, fumigation combined with intraperitoneal injection of CSE method to construct COPD animal

model, divided into control group, COPD group, ATG5$^{mye\triangle}$ group and ATG5$^{mye\triangle}$COPD group.A: morphological changes of lung tissue by HE staining in control group, COPD group, ATG5$^{mye\triangle}$group and ATG5$^{mye\triangle}$COPD group. compared with the control group, the wild COPD group and ATG5$^{mye\triangle}$COPD group showed broken and fractured alveolar walls, forming larger cavities, showing obvious emphysematous changes, the lung inflammatory cell infiltration was increased in ATG5$^{mye\triangle}$COPD mice compared with the wild-type COPD group mice (black arrows; magnification, ×400); B: changes in lung function-related indexes of mice in control group, COPD group, ATG5$^{mye\triangle}$group and ATG5$^{mye\triangle}$COPD group. n=3 experiments, $^{*}P < 0.05$, compared with the control group; $^{\&}P > 0.05$, compared with the control group; $^{**}P < 0.05$, compared with the COPD group; $^{\#}P < 0.05$, compared with the ATG5$^{mye\triangle}$group.

Figure4:

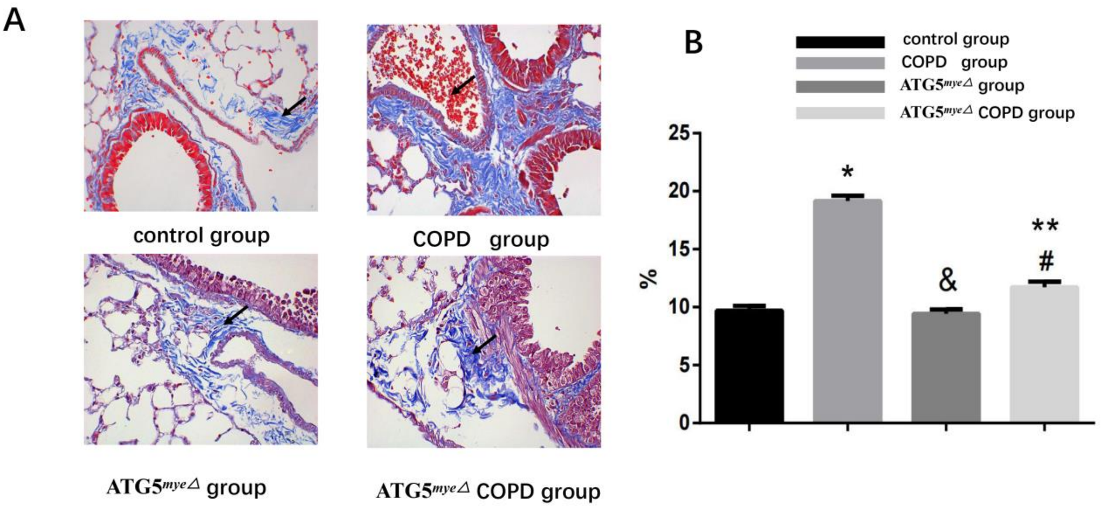

Figure 4: Effect of macrophage autophagy on small airway remodeling in COPD.Construction of ATG5 $^{mye\triangle}$mice, fumigation combined with intraperitoneal injection of CSE method to construct COPD animal model, divided into control group, COPD group, ATG5$^{mye\triangle}$ group and ATG5$^{mye\triangle}$COPD group. A: morphological changes of mouse lung tissue after masson staining in control group, COPD group, ATG5$^{mye\triangle}$group and ATG5$^{mye\triangle}$group (blue stain, black arrows; magnification, ×400); B: collagen volume fraction (CVF) after staining by masson staining method in control group, COPD group, ATG5$^{mye\triangle}$group and ATG5$^{mye\triangle}$group. n=3 experiments, $^{*}P < 0.05$, compared with the control group; $^{\&}P > 0.05$, compared with the control group; $^{**}P < 0.05$, compared with the COPD group; $^{\#}P < 0.05$, compared with the ATG5$^{mye\triangle}$group.

Figure5:

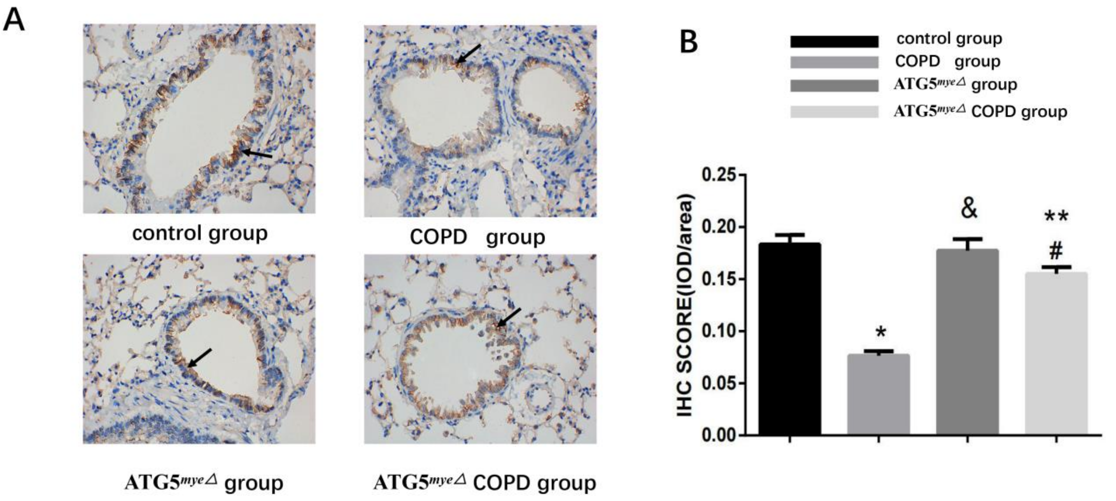

Figure 5: Effect of macrophage autophagy on expression of E-cadherinb in COPD.Construction of ATG5 $^{mye\triangle}$mice, fumigation combined with intraperitoneal injection of CSE method to construct COPD animal model, divided into control group, COPD group, ATG5$^{mye\triangle}$ group and ATG5$^{mye\triangle}$COPD group. A: changes in E-cadherin expression in lung tissues of mice in control group, COPD group, ATG5$^{mye\triangle}$group and ATG5$^{mye\triangle}$group(brown stain, black arrows; magnification, ×400); B: quantitative changes in E-cadherin expression in lung tissues of mice in control group, COPD group, ATG5$^{mye\triangle}$group and ATG5$^{mye\triangle}$group. n=3 experiments, *$P < 0.05$, compared with the control group; $^{\&}P > 0.05$, compared with the control group; **$P < 0.05$, compared with the COPD group; $^{\#}P < 0.05$, compared with the ATG5$^{mye\triangle}$group.

Figure6:

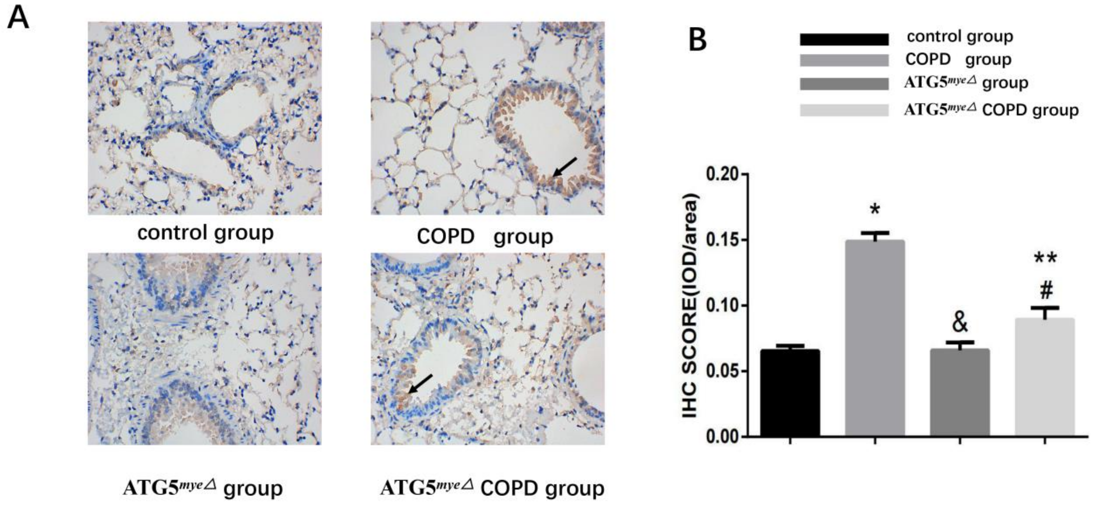

Figure 6: Effect of macrophage autophagy on expression of N-cadherinb in COPD.Construction of ATG5 $^{mye\triangle}$mice, fumigation combined with intraperitoneal injection of CSE method to construct COPD animal model, divided into control group, COPD group, ATG5$^{mye\triangle}$ group and ATG5$^{mye\triangle}$COPD group.A: Changes in N-cadherin expression in lung tissues of mice in control group, COPD group, ATG5$^{mye\triangle}$group and ATG5$^{mye\triangle}$group(brown stain, black arrows; magnification, ×400); B: Quantitative changes in N-cadherin expression in lung tissues of mice in control group, COPD group, ATG5$^{mye\triangle}$group and ATG5$^{mye\triangle}$group. n=3 experiments, *$P < 0.05$, compared with the control group;

$^{\&}$P > 0.05, compared with the control group; **P < 0.05, compared with the COPD group; $^{\#}$P < 0.05, compared with the ATG5$^{mye\triangle}$group.

Figure7:

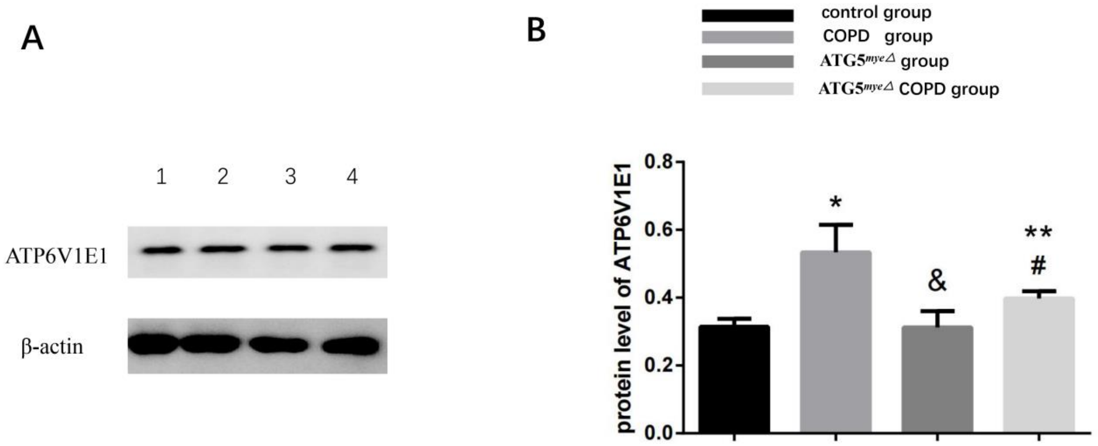

Figure 7: The expression level of ATP6V1E1 in COPD lung tissue, construction of ATG5 $^{mye\triangle}$mice, fumigation combined with intraperitoneal injection of CSE method to construct COPD animal model, divided into control group, COPD group, ATG5$^{mye\triangle}$group and ATG5$^{mye\triangle}$COPD group, Western blot method to determine the expression of ATP6V1E1 in lung tissue of each group. Western blot was used to determine the expression of ATP6V1E1 in lung tissue. A: changes in ATP6V1E1 expression in lung tissues of mice in control group, COPD group, ATG5$^{mye\triangle}$group and ATG5$^{mye\triangle}$COPD group; B: quantitative changes in ATP6V1E1 expression in lung tissues of mice in control group, COPD group, ATG5$^{mye\triangle}$group, ATG5$^{mye\triangle}$COPD group.n=3 experiments, *P < 0.05, compared with the control group; $^{\&}$P > 0.05, compared with the control group; **P < 0.05, compared with the COPD group; $^{\#}$P < 0.05, compared with the ATG5$^{mye\triangle}$group.

©2024 Zhang L. et al.
